# Comparison of clinical outcomes of hydrofiber and standard dressings in postoperative knee and hip arthroplasty wounds: a comprehensive meta-analysis

**DOI:** 10.3389/fsurg.2025.1684840

**Published:** 2025-12-12

**Authors:** Ran Zhang, Rui Sun

**Affiliations:** 1 Department of Joint Surgery, Tianjin Hospital, Tianjin, China; 2 Department of Thoracic Surgery, Tianjin Chest Hospital, Tianjin, China

**Keywords:** hydrofiber dressings, standard dressings, total knee arthroplasty, total hip arthroplasty, meta-analysis

## Abstract

**Objectives:**

The purpose of this study was to compare the clinical efficacy, safety and cost-effectiveness of hydrofiber dressings and standard dressings in the management of wounds after total knee or hip arthroplasty.

**Methods:**

This meta-analysis was conducted according the preferred reporting items for systematic reviews and meta-analyses (PRISMA) guidelines. We searched PubMed, EMBASE, and the Cochrane Library from inception to January 2025 to identify English-language randomized controlled trials (RCTs) and comparative cohort studies. Studies were included if they compared hydrofiber dressings with traditional or other standard dressings and involved patients undergoing primary hip or knee arthroplasty with an expected postoperative hospital stay of four days or more. The included studies were assessed for methodological quality. A meta-analysis and systematic review were performed on data extracted from these studies.

**Results:**

Nine RCTs with total 1,223 participants and three cohort studies with total 2,152 participants were included. The results showed that hydrofiber dressings significantly outperformed standard wound dressings in terms of blister formation (RR, 0.48; 95% CI, 0.27–0.87; *P* = 0.02; I^2^, 51%), periprosthetic joint infection (PJI) rate (RR, 0.27; 95% CI, 0.09–0.75; *P* = 0.01; I^2^, 0%), total complication rate (RR, 0.46; 95% CI, 0.29–0.75; *P* = 0.002; I^2^, 61%), number of dressing changes (MD, −1.87; 95% CI, −2.76 to −0.98; *P* < 0.0001; I^2^, 96%), and the need for dressing change within five days after surgery (RR, 0.55; 95% CI, 0.38–0.79; *P* = 0.001; I^2^, 43%). The systematic review showed that hydrofiber dressings were superior to standard wound dressings in terms of comfort and average total cost.

**Conclusion:**

The hydrofiber dressings were superior to standard wound dressings in terms of blistering, PJI rate, total complication rate, number of dressing changes, and need for dressing change within five days after surgery.

## 1 Introduction

Primary knee or hip arthroplasty is a common surgical procedure for patients with symptomatic end-stage osteoarthritis. One epidemiological study projected that the total number of primary knee arthroplasty (TKA) per year would reach 3.48 million by 2030 and that the total number of primary hip arthroplasty (THA) per year would reach 572,000 by 2030 (1). Although this method has better clinical outcomes than other treatment methods, it also has some complications, such as delayed wound healing, blister formation, and superficial or deep infection. The incidence of skin blistering is 13%–35% (2). Skin blisters can cause pain, discomfort, and postoperative morbidity, which may lead to prolonged hospital stays and even prosthetic joint infections. Infection is a significant complication of joint arthroplasty, often resulting from improper wound care. Reported infection rates for TKA range from 0.5% to 1.5% (3), while rates for THA fall between 1% and 2% (4). Such complications can necessitate prolonged intravenous antibiotics, increase medical costs, and elevate the rate of revision surgeries.

The risk of infection may increase as a result of slow wound healing. Thus, preventing complications and promoting optimal wound healing are important in postoperative care (5). Moist, clean, and warm environments have been shown to accelerate wound healing (6, 7). Consequently, an ideal dressing should effectively absorb excess exudate while maintaining a moist environment (6, 8). Moreover, a suitable dressing must create an impermeable barrier against external bacteria, while allowing the wound to breathe (9). However, the search for ideal dressings continues. Traditional gauze dressings are still widely used in most orthopedic centers, which often results in pain during removal, increased dressing changes, and prolonged healing times and hospital stays (10). Furthermore, traditional gauze dressings expose the aseptic wound to air, raising the risk of pathogen exposure, restricting patient self-care, and necessitating plastic bags or similar devices to keep the extremity dry (5).

To date, several types of modern wound dressings have been used in TKA and THA. These dressings vary in comfort level, cost, and purported efficacy in reducing wound complications. Hydrofiber dressings have gained popularity in orthopedic surgery due to their ability to absorb wound exudate into the dressing, transforming it into a gel and significantly increasing the volume of absorbable fluid. This process helps maintain a moist environment conducive to wound healing. Some products incorporate silver ions, which provide the dressing with antibacterial properties. Several randomized controlled trials (RCTs) have reported the benefits of hydrofiber dressings. Systematic reviews and meta-analyses (5, 11, 12) addressing optimal dressings for TKA have been published; however, the evidence presented was not sufficiently robust or precise. For example, Chen's systematic review compared adhesive and mesh dressings with other types but did not synthesize the data from the included studies (5). Mundi's study included all RCTs and cohort studies that compared hydrofiber dressings with standard dressings, which made the sample size was too small (11). Sharma's study compared various dressing materials, including hydrofiber, but did not assess them against standard dressings (12). Therefore, we conducted a comprehensive systematic review and meta-analysis to evaluate the clinical benefits and cost-effectiveness of hydrofiber dressings compared to other dressings, including traditional gauze dressings. We focused on the hydrofiber dressings and used the meta-analysis to get pooled results, which was more precise and robust than pre-studies (5, 11, 12).

## 2 Methods

This meta-analysis was carried out according to the guidelines of the Cochrane Collaboration but was not a formal Cochrane review (13). The findings are reported as recommended by the preferred reporting items for systematic reviews and meta-analyses (PRISMA) guidelines (14).

### 2.1 Eligibility criteria and search strategy

Studies were included if they involved adult patients undergoing primary hip or knee arthroplasty with an expected postoperative hospital stay of four days or more. Eligible study types included RCTs, prospective or retrospective cohort studies, and studies comparing hydrofiber dressings with traditional or other standard dressings. Studies were excluded if there were insufficient published data for meta-analysis studies, trials lacking a control group, non-peer reviewed sources, gray literature and literature published in a language other than English.

Three electronic databases (PubMed, EMBASE, and the Cochrane Library) were searched for relevant studies from their inception through January 26, 2025, using the keywords “hydrofiber dressing”, “dressing”, “Aquacel”, “knee arthroplasty”, “hip arthroplasty”, “knee replacement”, “hip replacement”, and “arthroplasty”. We also reviewed the references of included studies to ensure that all relevant studies were identified. No language restrictions were set.

### 2.2 Outcomes evaluated

The primary outcome was blistering. Secondary outcomes were redness of the skin, number of dressing changes, the need for dressing changes within five days after surgery, overall complication rate, infection rate, pain visual analog scale (VAS) pain, average total cost, and patient comfort.

### 2.3 Selection of studies and data extraction

The selection of studies was followed the PRISMA flow diagram. Duplicates were initially excluded using reference management software. During the title and abstract review stage, we excluded reviews, studies without a control group, and *in vitro* studies. Full-text screening was then performed on the remaining references by two independent reviewers, with any disagreements resolved through discussion until a consensus was reached.

Data extracted from included studies by two reviewers, using a standardized extraction table, included the first author, year of publication, country, study design, sample size, subject age, type of dressings, surgery type, intervention duration, and outcomes. We extracted means and standard deviations for continuous results.

### 2.4 Assessment of risk of bias for included studies

Two reviewers assessed the RCTs using a 12-item scale recommended by the Cochrane Back Review Group (15). Non-randomized controlled trials (non-RCTs) and cohort studies were assessed using the Newcastle–Ottawa Scale (NOS) (16). The NOS includes three domains: selection, comparability, and exposure, with a scoring system that ranges from zero to nine points. A score of more than six points was defined as high quality.

### 2.5 Data synthesis and sensitivity analysis

Mean differences (MD) with a 95% confidence interval (CI) were calculated to assess the effect size for continuous outcome data. A risk ratio (RR) with a 95% CI was used for dichotomous data. Statistical heterogeneity was assessed using the chi-square test and quantified with I^2^. All outcomes were analyzed using random effects models, irrespective of heterogeneity. A subgroup analysis was performed where feasible. A meta-analysis was conducted using RevMan 5.1. If data could not be synthesized, a descriptive analysis was performed.

If it was possible, we conducted the subgroup analysis according to the types of dressings to explain the heterogeneity from the differences of types of dressings.

Sensitivity analysis was conducted when three studies were included in the comparisons by omitting one or more studies that might increase the heterogeneity of each outcome. This was done to determine whether specific factors could influence the overall effects of each outcome.

The funnel plot was used to test for publication bias.

## 3 Results

### 3.1 Literature search and study characteristics

**Figure 1:**
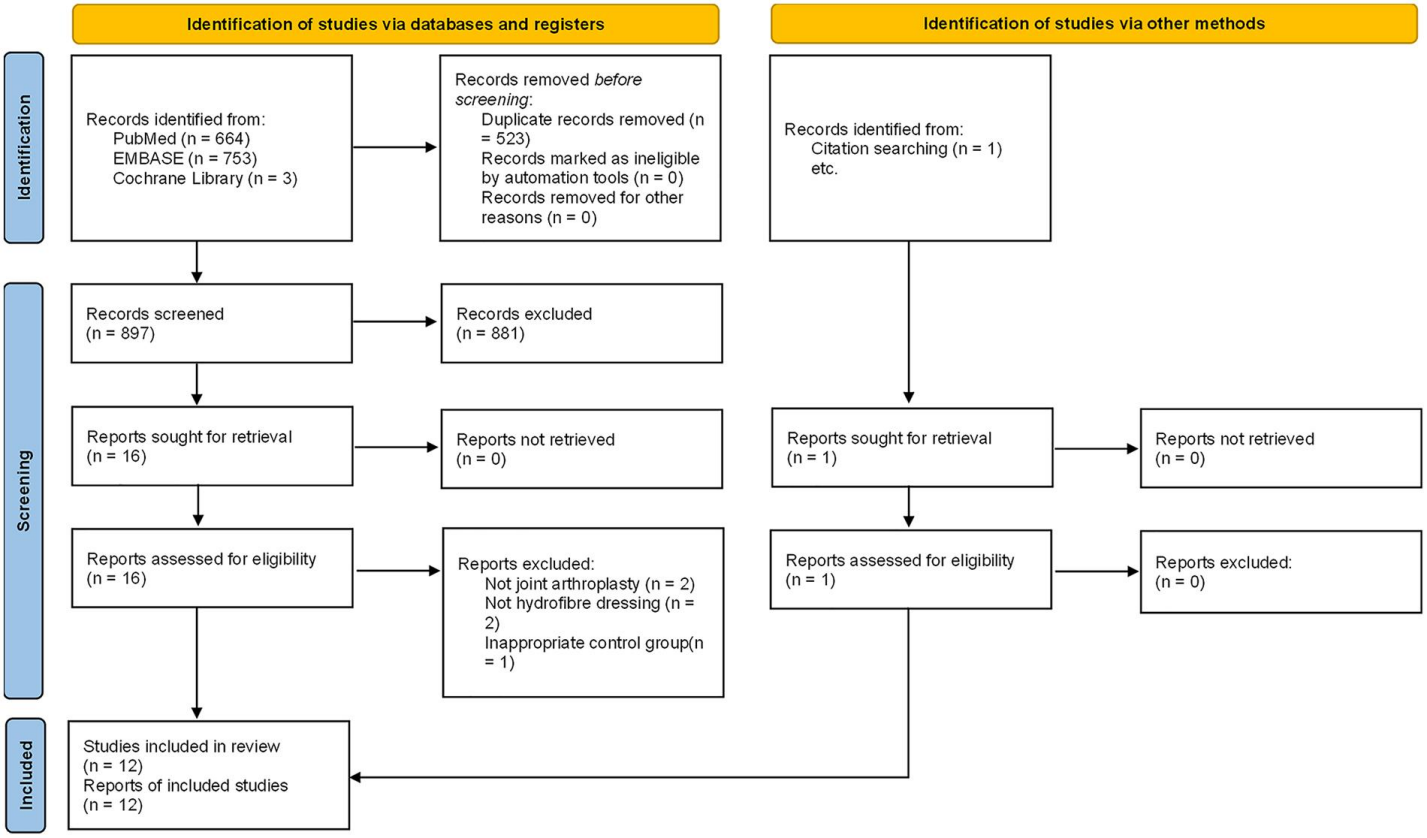
The literature selection flow diagram.

The selection flow of the studies is illustrated in Figure 1. The search strategy yielded 1,421 potential citations, with one retrieved from the reference list. After removing duplicates and scanning titles and abstracts, 17 citations remained for full-text review. Five trials were excluded for various reasons (Figure 1). Nine RCTs (17–25) and three non-RCTs (26–28) met the inclusion criteria and were included for synthesis or review. The total participants were 1223 in RCTs and 2125 in non-RCTs.

Table 1 summarizes the characteristics of the included RCTs and non-RCTs. The baseline demographic characteristics of four trials (17, 20, 21, 28) were unclear. One trial (26) included a small number of patients undergoing unicompartmental knee arthroplasty (UKA). All included trials utilized Aquacel® hydrofiber dressings, with four trials (23, 24, 27, 28) using silver-impregnated versions. Two trials (24, 27) reported the Zimmer NexGen, Legacy Posterior-Stabilized Prosthesis was applied to patients undergoing TKA. Three trials (22, 24, 27) reported that the wounds were closed with interrupted skin stitches. One trial (23) reported that a running 3-0 Monocryl stitch was placed in the subcuticular layer and was followed with a skin adhesive. Hopper's study (26) used staples or subcuticular sutures. Five studies (19, 22–24, 27) reported using the suction drain. No included trials reported the solution that were used to disinfect the wounds.

**Table 1:** The characteristics of included studies.

Study	Study design	Country	Sample size		Age		Surgery type	Interventions		Intervention duration	Outcomes
			Hydrofibre dressing	Standard dressing	Hydrofibre dressing	Standard dressing		Hydrofibre dressing	Standard dressing		
Harle 2005	RCT	Finland	50	50	65 (13.5)	64.3 (11.8)	THA	Aquacel dressing	Wound pad dressing	2 months	Complications, cost
Abuzakuk 2006	RCT	UK	30	31	69.6 (10.9)	70.1 (10.8)	TKA/THA	Aquacel dressing	Central pad (Mepore) dressing	5 days	Complications, Change dressing, patient comfort
Ravenscroft 2006	RCT	UK	85	98	NR	NR	TKA/THA/hemi-HA/DHS	Aquacel dressing + Tegaderm	Cutiplast	3 months	Dressing failed, wound healed, complications, number of dressing change, VAS
Burke 2012	RCT	Ireland	62	62	67		TKA/THA	Jubilee dressing (Aquacel + DuoDerm)	Central pad (Mepore) dressing	NR	Complications, number of dressing change, cost
Dobbelaere 2015	RCT	Belgium	29	31	63.4 (23–88)		TKA	Aquacel dressing	Cosmopor E	5 days	Complications, dressing change, cost, VAS
Langlois 2015	RCT	France	40	40	71.1 (11.4)	68.3 (10.3)	TKA/THA	Aquacel dressing	Central pad (Mepore) dressing	6 weeks	Complications, number of dressing change, patient and nurse satisfaction, scar cosmetic appearance
Springer 2015	RCT	USA	141	121	62.09 (12.09)	62.92 (10.25)	TKA/THA	Aquacel Ag^+^ dressing	Primapore	4 weeks	Complications, number of dressing change, patient satisfaction
Kuo 2017	RCT	China	120	120	70.3 (7.5)	70.1 (7.1)	MIS-TKA	Aquacel Ag^+^ dressing	Sofra-Tulle dressing	NR	Complications, number of dressing change, VAS, patient comfort
Beele 2020	RCT	Belgium	57	56	68.5 (11.2)	69.2 (9.8)	TKA/THA	Aquacel dressing	Mepilex Border Post-Op	5 days	Complications, dressing failed, VAS, dressing change, patient and nurse satisfaction
Hopper 2012	PCS	UK	50	50	67.7 (51–83)	68.8 (45–86)	TKA/THA/UKA	Aquacel dressing	Central pad (Mepore) dressing	6 weeks	Complications, number of dressing change
Cai 2014	RCS	USA	903	875	NR	NR	TKA/THA	Aquacel Ag^+^ dressing	Xeroform and gauze with ace bandage	NR	PJI
Akdoğan 2020	RCS	Turkey	139	135	67.6 (7.6)	65.4 (5.7)	TKA	Aquacel Ag^+^ dressing	Conventional gauze sponge	NR	Complications, number of dressing change, VAS, patient satisfaction

RCT, randomized controlled trial; PCS, prospective cohort study; RCS, retrospective cohort study; TKA, total knee arthroplasty; THA, total hip arthroplasty; UKA, unicompartmental knee arthroplasty; NR, not report; VAS, Visual Analogue Scale; PJI, periprosthetic joint infection.

### 3.2 Quality assessment of studies

In the RCTs, the methodological quality was high for three trials (22, 24, 25), moderate for four trials (17–19, 23), and low for two trials (20, 21). One trial (21) did not implement proper randomization, while two trials (22, 25) employed blinding of outcome assessors (Table 2).

**Table 2:** Methodological quality assessment of included randomized controlled trials.

Included studies	Randomized adequately^a^	Allocation concealed	Similar baseline	Patient blinded	Care provider blinded	Outcome assessor blinded	Avoided selective reporting	Similar or avoided cofactors	Patients’ compliance^b^	Acceptable drop-out rate^c^	Similar timing	ITT^d^ analysis	Quality^e^
Harle 2005	Yes	Yes	Yes	No	No	No	Yes	Yes	Unclear	No	Yes	No	Moderate
Abuzakuk 2006	Yes	No	Yes	No	No	No	Yes	Yes	Unclear	Yes	Yes	No	Moderate
Ravenscroft 2006	Yes	No	Unclear	No	No	No	Yes	No	Unclear	Yes	Yes	No	Low
Burke 2012	Yes	No	Unclear	No	No	No	Yes	Yes	Unclear	Yes	Yes	No	Moderate
Dobbelaere 2015	Unclear	No	Unclear	No	No	No	Yes	No	Unclear	Yes	Yes	No	Low
Langlois 2015	Yes	No	Yes	No	No	Yes	Yes	Yes	Unclear	Yes	Yes	No	High
Springer 2015	Yes	No	Yes	No	No	No	Yes	No	Unclear	Yes	Yes	No	Moderate
Kuo 2017	Yes	Yes	Yes	No	No	No	Yes	Yes	Unclear	Yes	Yes	Yes	High
Beele 2020	Yes	No	Yes	No	No	Yes	Yes	Yes	Unclear	Yes	Yes	Yes	High

a Only if the method of sequence generated were explicitly described could get a “Yes”; sequence generated by “dates of admission” or “patient number” receive a “No”.

b Intermittent treatment or therapeutic duration less than 6 months means “Yes”, otherwise “No”.

c Drop-out rate ≥30% means “No”; <30% means “Yes”.

d ITT, intention to treat, only if all randomized patients are analyzed in the group they were allocated to could receive a “Yes”.

e The frequencies of “Yes” ≥7 means “High”; ≥4 and <7 means “Moderate”; ≤4 means “Low”.

In the non-RCTs, one trial (26) achieved a score of 8, one achieved 7 (27), and one achieved 5 (28) (Table 3).

**Table 3:** The methodological quality assessment of non-RCTs.

Included studies	Selection	Comparability	Exposure	Total score
Hopper 2012	4	1	3	8
Cai 2014	2	0	3	5
Akdoğan 2020	3	2	2	7

### 3.3 Blisters

**Figure 2:**
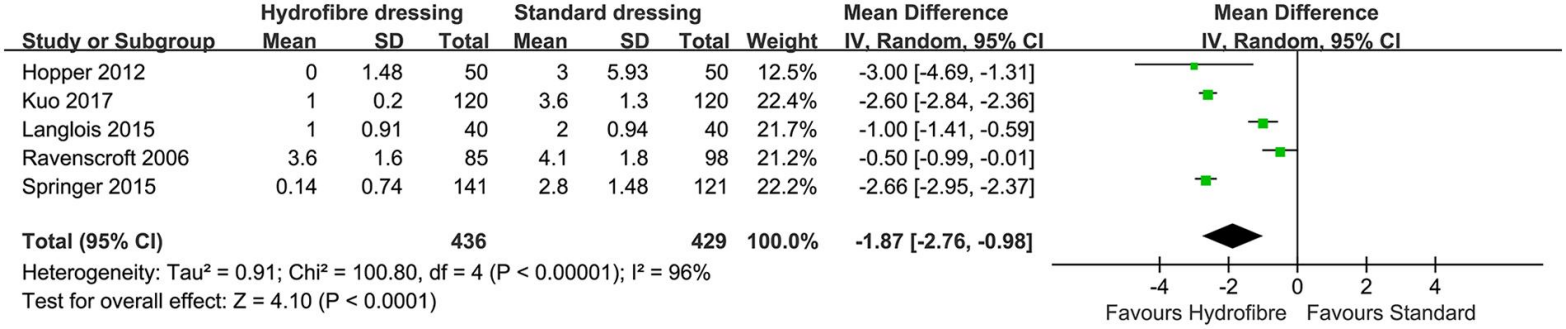
The forest plot of blisters between hydrofiber dressings and standard dressings.

All nine RCTs (17–25) and two non-RCTs (26, 27) reported the risk of wound blisters and were included in the pooled analysis. Subgroup analysis showed a statistically significant decrease in the odds of blisters for hydrofiber dressings in RCTs (RR, 0.47; 95% CI, 0.22–0.98; *P* = 0.04; I^2^, 54%). Subgroup analysis according to Mepore dressings also showed a statistically significant decrease for hydrofiber dressings in RCTs (RR, 0.39; 95% CI, 0.18–0.85; *P* = 0.02) and the heterogeneity was low (I^2^, 0%). In non-RCTs, there was no significant difference between the two groups (RR, 0.48; 95% CI, 0.11–2.07; *P* = 0.33; I^2^, 69%; Figure 2). However, the overall effect test favored the hydrofiber dressings (RR, 0.48; 95% CI, 0.27–0.87; *P* = 0.02; I^2^, 51%; Figure 2). The results of this comparison remained unchanged after sensitivity analysis.

### 3.4 Infection rate

**Figure 3:**
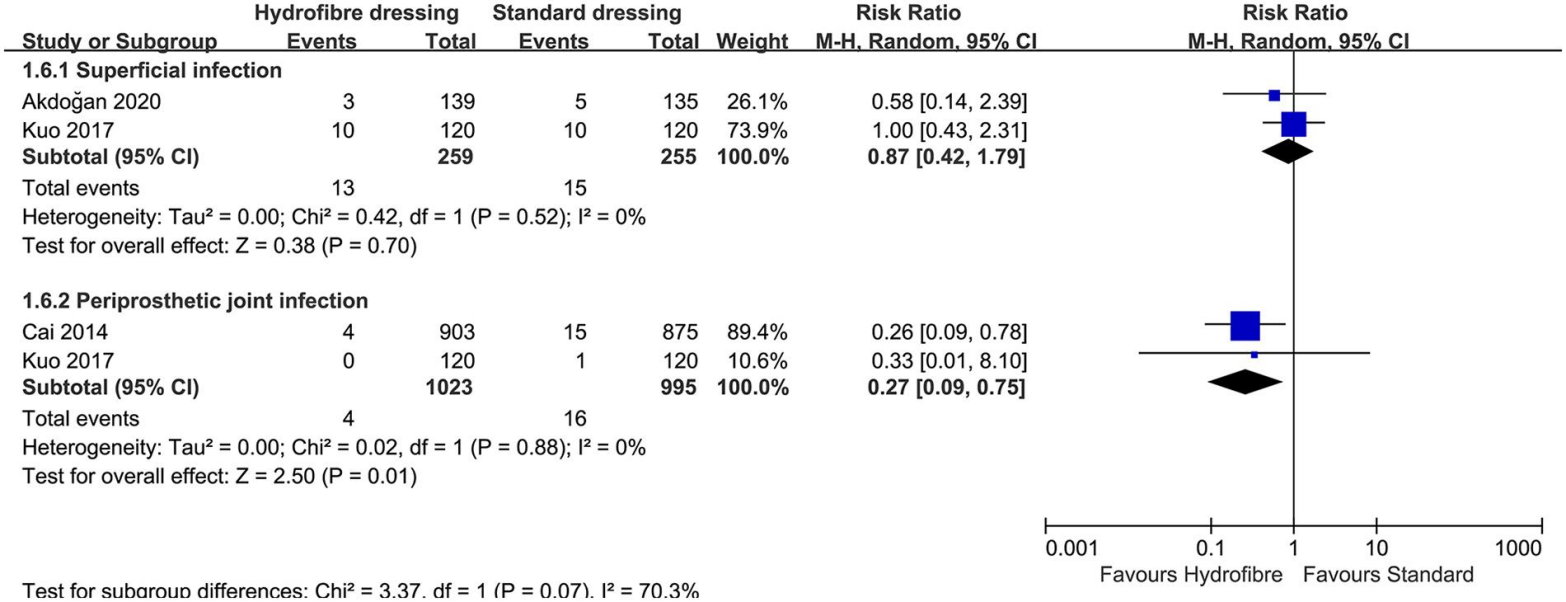
The forest plot of infection rate between hydrofiber dressings and standard dressings.

Five trials (17, 21, 24, 27, 28) reported infection rates. However, two studies (17, 21) had double zero events for superficial infection rates and could not be used for pooled analysis. Among these trials, one (27) had a superficial infection rate, one (28) had a PJI rate, and the remaining trial (24) reported both superficial and periprosthetic joint infection rates. There was no significant difference in the superficial infection rate in the pooled treatment effect (RR, 0.87; 95% CI, 0.42–1.79; *P* = 0.70; I^2^, 0%). However, there was a significant reduction in the PJI rate with the use of hydrofiber dressings (RR, 0.27; 95% CI, 0.09–0.75; *P* = 0.01; I^2^, 0%; Figure 3). Sensitivity analysis was not performed due to the small number of included studies.

### 3.5 Overall complication rate and redness of the skin

Four (18, 20, 22, 23) of the 12 studies reported a total complication rate. The pooled effect estimate showed a statistically significant reduction in the total complication rate with the use of hydrofiber dressings (RR, 0.46; 95% CI, 0.29–0.75; *P* = 0.002; I^2^, 61%). The results of this comparison remained unchanged after sensitivity analysis.

Five RCTs (17, 18, 21, 22, 25) reported redness of the skin. The pooled effect estimate showed no significant difference between the two groups for this outcome (RR, 0.21; 95% CI, 0.21–1.33; *P* = 0.18; I^2^, 62%). Sensitivity analysis showed that the overall effect could be affected by omitting the Beele's study (25). Because the dressings used in Beele's study (25) and Dobbelaere's study (21) were different from other three included studies, when we omitted the two studies, the heterogeneity was significantly decreased (I^2^, 13%), and the pooled result showed that hydrofiber dressings significantly decrease the rate of redness of the skin (RR, 0.36; 95% CI, 0.17–0.75; *P* = 0.006).

### 3.6 Number of dressing changes

**Figure 4:**
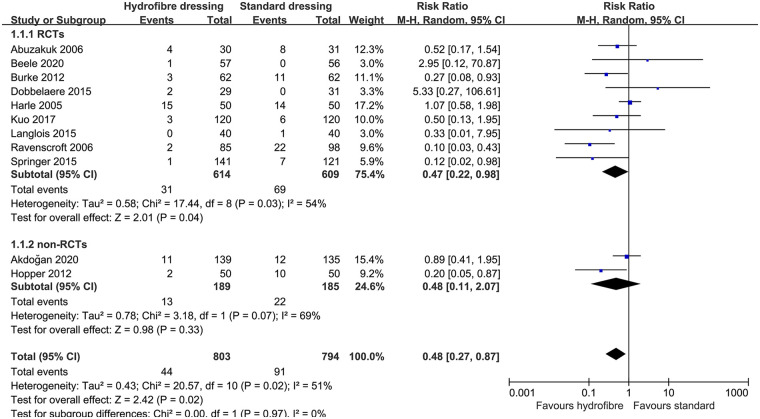
The forest plot of number of dressing changes between hydrofiber dressings and standard dressings.

Five studies (20, 22–24, 26) reported the number of dressing changes. The pooled effect showed that hydrofiber dressings significantly reduced the number of dressing changes compared to the other wound dressings (MD, −1.87; 95% CI, −2.76 to −0.98; *P* < 0.0001; I^2^, 96%; Figure 4). The results of this comparison remained unchanged after sensitivity analysis.

### 3.7 Need for dressing change within five days after surgery

Four trials (17–19, 25) reported the need for dressing changes within five days after surgery. The pooled effect indicated that hydrofiber dressings had a statistically lower need for dressing change within five days post-surgery compared to the control group (RR, 0.55; 95% CI, 0.38–0.79; *P* = 0.001; I^2^, 43%). However, sensitivity analysis revealed that the overall effect could be influenced by omitting either the Abuzakuk or Harle study. Because the dressings used in Beele's study (25) was different from other three included studies, when we omitted this study, the heterogeneity was significantly decreased (I^2^, 24%), and the pooled result did not change (RR, 0.51; 95% CI, 0.38–0.69; *P* < 0.0001).

### 3.8 VAS

Six trials (20–22, 24, 25, 27) reported the VAS. However, the data of two (21, 27) of the six trials were not intact, and the measurement range of the VAS of one trial (25) was different from the others. There was no significant difference in VAS between the two groups (MD, −1.07; 95% CI, −2.80–0.66; *P* = 0.23; I^2^, 99%). The results of this comparison remained unchanged after sensitivity analysis.

### 3.9 Comfort level

Five trials (19, 22–24, 27) reported the patient evaluation of comfort level. Three trials (23, 24, 27) reported that the comfort level of hydrofiber dressings was significantly higher than that of traditional gauze dressings. Two studies (19, 22) reported no significant differences between the two groups. The control group for these two studies used central pad dressings.

### 3.10 Average total cost

Three trials (17, 18, 21) reported the cost of dressings. All included trials indicated that the average total costs of hydrofiber dressings were higher than those of conventional dressings.

### 3.11 Publication bias assessment

**Figure 5:**
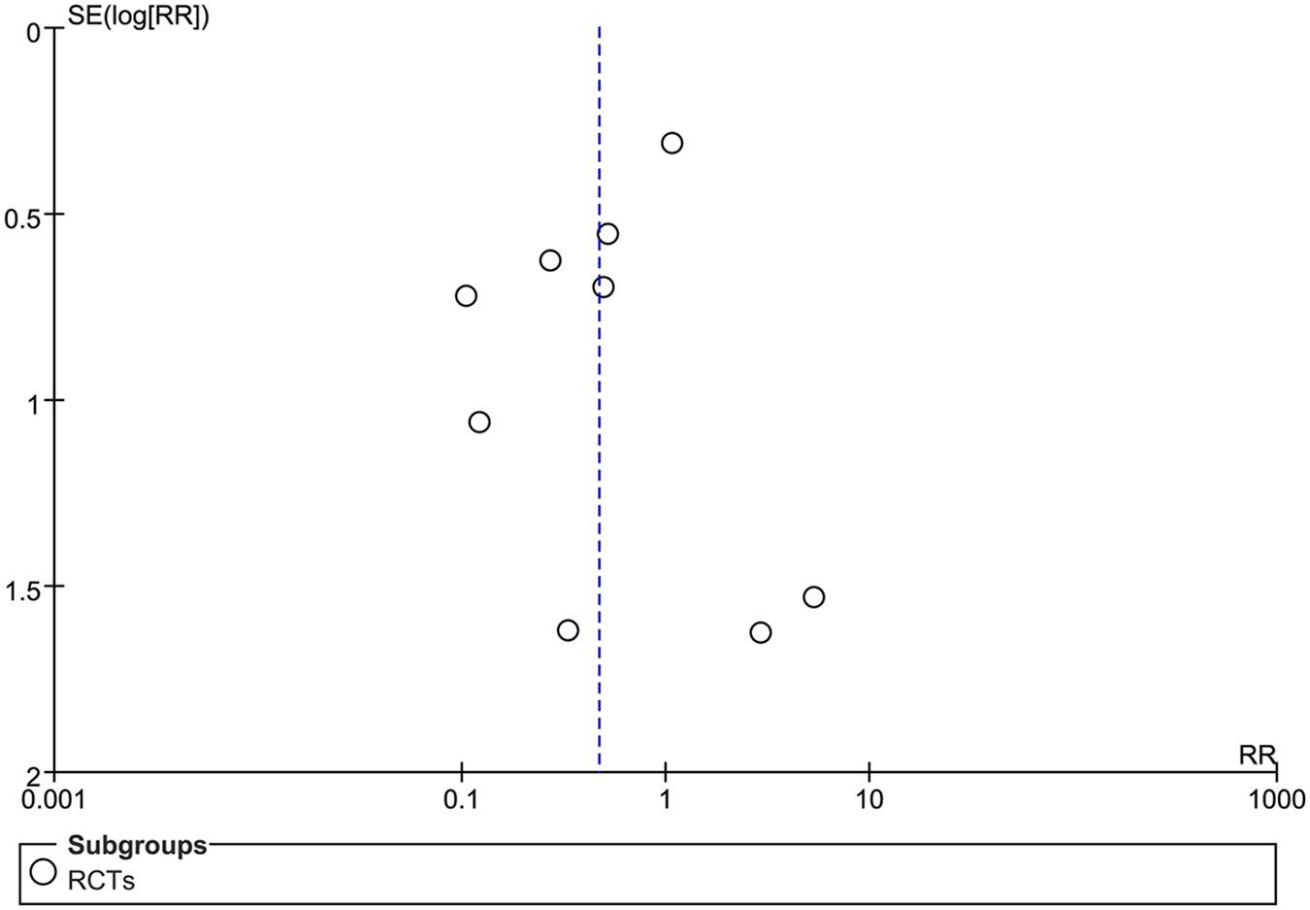
The publication bias of RCTs.

Funnel plot indicated the blisters in included studies is shown in Figure 5. The shape of the funnel plot reveals slight asymmetry, which indicated some evidence of publication bias.

## 4 Discussion

Wound care is important for rehabilitation after TKA or THA. Hydrofiber dressings have been shown to be effective in pressure sores, leg ulcers and burn wounds (29, 30). Other study recommended the use of hydrofiber dressings on open acute surgical wounds. Our meta-analysis was similar to these studies, which might because hydrofiber dressings could produce warm, moist, local wound conditions for optimum wound healing.

Blisters can easily lead to skin breakdown, which may provide an entry point for wound infections or PJIs (23). Factors such as swelling after surgery, friction between the dressing and skin, and dry, hard dressings may cause separation of the epidermis from the dermis. Therefore, the extensibility of the wound dressing should accommodate changes in wound length during joint flexion to minimize friction. The dressing should maintain softness after absorbing wound exudate. Hydrofiber dressings come in various sizes, conform to body contours, and can be layered during application, which makes them more suitable for skin extensibility. The Aquacel® dressing quickly converts into a soft gel upon absorbing wound exudate, which provides a gentle environment for the wound (31). Abuzakuk et al. (19) noted that wound blisters required additional dressing changes, increased nursing time, delayed patient discharge, and increased total costs. Our meta-analysis confirmed this finding. Reducing the blister rate may decrease the incidence of superficial or periprosthetic infections, shorten hospital stays, and lower total costs.

One study emphasized that maintaining a moist environment is key to healthy wound healing (32). Several studies have demonstrated that a moist environment can accelerate wound healing (6–8). A study by Field and Kerstein (33) confirmed that moist wounds experience less pain, fewer infections, and faster healing compared to dry wounds. Aquacel® autolytic debridement dressing, composed of 100% sodium carboxymethylcellulose with no active ingredients, was the intervention in all included trials in our study. Sodium carboxymethylcellulose is the primary ingredient in hydrocolloid dressings. Aquacel® dressings absorb wound exudate into the fibers, which significantly increases the volume of exudate they can hold. When wound exudate emerges, the dry Aquacel® quickly converts into a soft gel sheet that covers the wound surface and maintains warm, moist wound conditions optimal for healing (31). This effect may reduce irritation and maceration.

As analysis above, hydrofiber dressing could reduce pain on wound. However, the VAS result of our meta-analysis was not significant. This might be result from small number of included studies or high heterogeneity among included studies which might be result from different types of dressings in control group.

There was no significant difference in redness of the skin in our meta-analysis. However, we conducted the subgroup analysis. When omitted the studies with different types of dressings in control group, the heterogeneity was reduced, and the pooled result changed to significant favor the hydrofiber dressings.

Cost-effectiveness is an important consideration in wound care. Several studies (34, 35) have reported that hydrofiber dressings can be worn for longer periods, resulting in a reduction in the number of wound dressing changes. Although hydrofiber dressings are more expensive than traditional dressings, this cost is offset by the reduced frequency of dressing changes. Our study found that both the number of dressing changes and the need for dressing change within five days after surgery were statistically lower for hydrofiber dressings than for other wound dressings, which is consistent with the conclusion of the above study. However, if the cost of hydrofiber dressing was too high in some countries, the superiority of hydrofiber dressings might be offset.

Five included studies (18, 20, 22, 23, 26) reported follow-up periods exceeding four weeks. Most studies did not provide detailed descriptions of the dressings used for wounds after discharge. Nurses or doctors typically evaluated the wound and surrounding skin, with some studies offering suggestions for dressing changes. This may have influenced the trial results.

The heterogeneity was high in some outcomes of this meta-analysis. We used subgroup analysis according to type of dressings if possible. We found that five included studies (17–19, 22, 26) were used the wound pad dressing as the control group and the main source of heterogeneity was types of dressings. However, among the outcomes which conducted the subgroups analysis, only the pooled result of redness of the skin was changed. More same control group studies were needed to reduce the heterogeneity for this topic.

### 4.1 Limitations

Our meta-analysis and systematic review have several limitations. First, several nontraditional dressings were compared with hydrofiber dressings, resulting in clinical heterogeneity. However, we used a random-effects model to analyze each outcome, which helps minimize the effect of clinical heterogeneity. Second, our study included three non-RCTs, which may lower the level of evidence. Nevertheless, we performed a subgroup analysis to determine whether it was possible to reduce affection. Therefore, these results should be interpreted with caution. Third, there was no standardized method for measuring comfort, so the findings of this systematic review should also be interpreted cautiously. Fourth, the kind of prosthesis, types of suture for closing the wound, drainage and solution used for disinfection might affect the results. However, we could not conduct subgroup analysis according to these factors. Lastly, the small number of studies might reduce the statistical power.

## 5 Conclusion

Our meta-analysis and systematic review indicated that hydrofiber dressings significantly outperform standard wound dressings in terms of blisters, PJI rates, total complication rates, number of dressing changes, and the need for dressing changes within five days after surgery. Based on these findings, we recommended hydrofiber dressings for postoperative wounds in TKA or THA due to their advantages in wound management. However, further well-designed RCTs with larger participant numbers, long-term follow-ups, and standardized evaluation criteria are needed to improve the level of evidence regarding comfort level and average total cost. Future research should also compare hydrofiber dressings with other modern dressing options.

## Data Availability

The original contributions presented in the study are included in the article/Supplementary Material, further inquiries can be directed to the corresponding author.
